# Frequency, topic, and preferences: Tracking student engagement with several modalities of student–instructor contact in a first‐year course

**DOI:** 10.1002/2211-5463.13315

**Published:** 2021-10-31

**Authors:** Bailey E. Bingham, Victoria Rea, Lisa Robertson, M. Alex Smith, Shoshanah Jacobs

**Affiliations:** ^1^ Department of Integrative Biology University of Guelph ON Canada; ^2^ Department of Cellular and Molecular Biology University of Guelph ON Canada; ^3^ CBS Office of Educational Scholarship and Practice University of Guelph ON Canada

**Keywords:** undergraduate biology, office hours, student–instructor contact, contact preferences, workload equity

## Abstract

Meaningful student–instructor interactions during an undergraduate degree course can have important effects on student learning. The format by which those interactions are made possible can vary greatly. We investigated the preferred modality of contact and students’ reasons for contact across several modalities in a first‐year biology course. We tracked student–instructor contact for two‐course instructors who team teach collaboratively (rather than sequentially) across two‐course sections. Both instructors had identical scores on student evaluations of approachability. Student–instructor contact was facilitated using five ‘student hour’ modalities: (a) in office by appointment, (b) 1 h per week, in office drop in, (c) 1 h per week, virtual chat, (d) by email, (e) 10 min immediately after class. Though email was the preferred method of contact, the period immediately following the class instruction was the most popular of the face‐to‐face options. We note significant differences in the distribution of workload across the two instructors and make recommendations for increasing the accessibility of student–instructor contact and for equity in workload to support student learning.

## Graphical abstract

We conducted a study to determine which of the available means of student‐instructor contact were most preferred by students. Email was the most popular means of contact, followed by in‐person contact immediately after class. We observed that the number of student contact events differed among the three members of the teaching team. Recommendations are provided for student‐centered approaches to teaching in higher education.

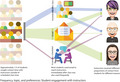

**Abbreviations**
LMS: learning management system

Interacting with instructors is an integral component of a student’s experiences in post‐secondary education. Student–instructor interactions are consistently linked with positive outcomes, including academic success [1], retention and persistence [2], academic motivation [3], academic self‐concept [4], and general well‐being [5]. Furthermore, the effects of student–instructor interactions may be especially beneficial for minority student groups [6, 7, 8, 9, 10]. As such, student–instructor interaction is one of the most empirically supported educational best practices in higher education [11].

However, the frequency of student–instructor interactions alone is not enough to ensure positive student outcomes [12, 13], as the quality of these interactions is an important mediating factor [5, 14, 15, 16]. For instance, relationships with instructors that are based on respect, support, and mentorship predict greater positive outcomes for students, including increased confidence [17], academic motivation [13], engagement [6], and performance [17]. Similarly, academically challenging and/or research‐oriented interactions are especially impactful for students’ academic motivation and aspirations for further study [3, 13].

Despite the robust evidence for the value of high‐quality student–instructor interactions, many studies have found that these interactions are infrequent outside of class time [18, 19, 20, 21]. Office hours are one institutional practice that are designed to provide opportunities for such interactions. Yet, office hour offerings are consistently under‐accessed by students [15, 18, 22]. Little research exists to outline best practices for office hour implementation which reduce barriers to access and promote more frequent, high‐quality interactions between students and instructors.

Lack of convenience and instructor unapproachability are cited as major factors limiting student engagement with office hours [15, 18, 23, 24] and students indicate that they prefer to communicate with instructors by email or in brief interactions before and after class [20, 23]. However, it is unlikely that these brief interactions represent meaningful contact with instructors or support the associated positive outcomes [12, 19]. In this study, we aim to identify best practices for the implementation of office hours which support high‐quality interactions between instructors and students. Specifically, we track the frequency and topic of student–instructor interactions across several formats (including virtual and in‐person office hours, appointments, email, and visits before/after class) in a first‐year biology course. This course is co‐taught by two tenured faculty, and therefore, we present findings in the context of a team‐teaching approach where differences were perceived by students with respect to personality traits and gender/sex presentation. Though not a controlled study on these differences, workload distribution in team‐teaching courses is generally considered in the context of these differences.

## Literature review

### Modes of student–instructor interaction

Frequent and high‐quality, student–instructor interactions are one of the most empirically supported educational best practices in higher education [11] and have been connected to substantial benefits to students. Office hours, whereby instructors commit between one and several hours weekly to meeting informally with students, are an institutionally supported practice designed to promote student–instructor interactions. Yet, office hour offerings are consistently under‐accessed by students [15, 18, 22]. Factors related to scheduling, convenience, and instructor availability are all cited as significant barriers to student engagement with office hours [15, 22, 23, 24]. Meetings by appointment and virtual office hour offerings both represent alternatives to the traditional format which may alleviate some barriers, while establishing others. However, engagement with alternative forms of office hours has been mixed [23], and though students indicate greater course satisfaction when office hours are offered virtually compared to in‐person formats, student engagement with virtual office hours is still low [20, 23].

Student avoidance of office hours in their various formats supports the notion that there is a mismatch between their intended and perceived purpose. While office hours are designed to provide an opportunity for interactions which are meaningful and supportive of student success, students are either unsure of the purpose of office hours, or view office hours as opportunities for students in crisis to contact the instructor [15]. Students indicate that they prefer email or talking after class as their primary way of communicating with their instructor [15, 20, 23] though it is unlikely that these brief and/or asynchronous communications support high‐quality interactions. Additional research is needed to discern the amount that these various formats promote meaningful student–instructor contact.

### Topic of interaction

The quality of student interactions with instructors is an important mediating factor for effecting positive student outcomes [3, 5, 13, 14, 15, 17]. Yet, student–instructor interaction is often measured by the frequency of student engagement and not by the quality [9, 20]. This quantitative approach provides insight into the methods and formats by which students interact with instructors, but it obscures the variation in the meaningfulness of these interactions.

Pascarella & Terenzini (1991) outlined the importance of both the frequency and the nature of interactions, theorizing that substantive topics such as research or career development posed greater benefits for students. Many of the existing scales of student–instructor interactions align with this typology, categorizing interactions based on their substantive focus [9, 12, 13, 16, 25]. Interactions whereby students volunteer or work on research with a faculty member are frequently differentiated from interactions which are related to course work, for example [12, 13]. Other researchers differentiate based on the purpose of an interaction, distinguishing logistical queries and content‐related questions from discussions of theory and applications of learning [19, 25]. The psychosocial qualities of student–instructor interactions have also been examined, with several researchers categorizing personal interactions and interactions that involve support and mentorship as distinct types of student–instructor contact [5, 16, 19]. Despite the variation in approaches to measurement, the value of high‐quality, student–instructor interaction is well supported. Therefore, the practices that best support these meaningful interactions warrant additional study.

### Impact of instructor personality traits and presentation on student engagement

It is well established that students in higher education have preferences for certain instructors based on a number of visible and non‐visible factors [26, 27, 28]. Thus, there are likely to be disparities in co‐teaching teams, resulting in more interaction with certain instructors that may result in greater workloads. A recent study by Clayson (2019) found that instructor age, gender, and political leaning had an effect on how much students anticipate the instructor being helpful and how much they expect to learn from that instructor. A study examining student reports of qualities in an ideal instructor revealed that students prefer instructors to have certain personality traits at higher levels than both themselves and the general population but that students prefer instructors with personality traits that they perceive as similar to themselves [27]. Students also favor emotional stability and conscientiousness, which may be perceived by students as early as the first class [27, 29]. Another report suggests that students prefer instructors with high professional knowledge of the field in which the course is based [30] and perceived approachability of the instructor may play a role in student use of office hours [15]. Women are often viewed as more approachable [31] and previous research indicates that many students with personal problems or requests for favors prefer to contact female instructors [32]. However, it is unclear how the gender/sex presentation of the instructor relates to student engagement with office hours and other formats of student–instructor interaction. Understanding the conditional effects of instructor personality traits and gender/sex on how students initiate contact with their instructors is important for both parties.

### Purpose of study

Our study aims to identify best practices for implementing office hours which promote frequent, high‐quality interactions between instructors and students. We compare the frequency and general topics of student–instructor interaction across several commonly used formats. We also examine how instructor gender/sex presentation predicts student interactions, which has implications for both the value of a diverse teaching team and the relative workload related to student contact for each instructor. We aim to address two research questions:

1. Which format of contact with instructors supports the highest frequency of higher‐quality (as indicated by topic of inquiry) student–instructor interaction?
2. How might the personal qualities of the instructor impact the frequency and topic of student–instructor interactions?

Frequency and topic of student–instructor contact were tracked across five formats (in‐person office hours, appointments, visits before/after class, online instant messaging, and email) for two instructors and one course coordinator in two sections of a co‐taught, first‐year biology class.

## Methods

### Ethical statement

The teaching team from whom data were collected are also authors on this article and so written consent was not required. Because we predicted that student knowledge of the study might reduce their learning experience in the course, and because the detail level of information collected from students in the course did not require us to seek informed consent, we obtained ethical clearances for research involving human subjects without informed consent from the University of Guelph Research Ethics Board (REB# 19‐05‐022).

### Description of the course and the comparability of the instructors

We collected data from the teaching team of a first‐year, introductory biology course at the University of Guelph, Ontario (a comprehensive research and teaching university with approximately 26,000 undergraduate students), during the January–April 2020 (W20) semester. ‘Discovering Biodiversity’ (BIOL 1070) is a large, introductory class which focuses on evolution, ecology, and physiology and is taken by most students within the College of Biological Science during their degree. In W20, BIOL 1070 was delivered as two‐course sections, accommodating 445 students in section 01 and 440 students in section 02. Each section enrolled different students from similar programs (e.g., Biomedical Science, Human Kinetics), who were taught the same content by the same instructors. The sections themselves are the result of classroom maximum capacity rather than differences in student cohorts or course content. Students were able to choose which section they preferred based upon their personal schedules. The sections are taught ‘back to back’ and repeated near identically by the same instructors. The teaching team includes a course coordinator (LR) to manage small‐group seminars and course logistics, and a pair of instructors who co‐teach each of the two sections together (SJ and MAS). Both instructors were involved in the delivery of the course content and taught in a conversational style with frequent and improvised transitions between themselves throughout each lecture section. The course coordinator managed all the seminar tutorials, the teaching assistant team, and all incoming emails to the course‐designated email address. All students are asked to address all inquiries to the course‐designated email address to ensure a consistent and speedy reply.

Both instructors are associate professors within the Department of Integrative Biology (Table 1). One instructor had taught the course 8 times previously, and the other was teaching it for the first time, though they had audited the course and developed approximately 40% of the ‘flipped’ content over 6 years. Student evaluations, where students used a 5‐point scale to evaluate 12 measures of effective course design and instruction, were also administered in the W20 semester and these evaluations further supported the comparability of the two instructors. On a question of approachability of the instructors, MAS received a mean score of 4.44/5.00 and 4.52/5.00, and SJ received a near‐identical mean score of 4.45/5.00 and 4.52/5.00 from each of sections 01 and 02, respectively. One perceptible difference between the instructors is their sex presentation. We attempted to account for this and other differences between the two instructors by employing a switching replication design, whereby in course section 01 one instructor advertised scheduled office hours, while the other was accessible by appointment only, and then reversing the roles in course section 02.

**Table 1:** Attributes of the teaching team for Discovering Biodiversity (BIOL*1070) in the January–April 2020 semester.

	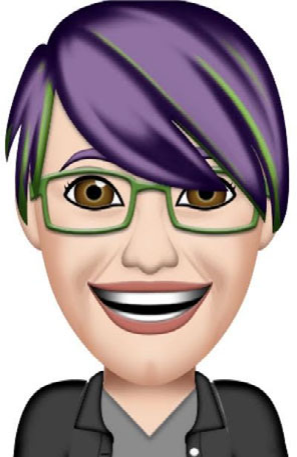 Instructor 1	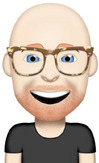 Instructor 2	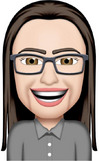 Course Coordinator
Characteristics that students could readily observe			
Sex presentation	Female	Male	Female
Age (years above youngest)	0	+6	0
Race	White	White	White
Style	Trendy	Sporty	Classic
Characteristics shared with students			
Role	Dr. Shoshanah Jacobs Instructor Associate Professor	Dr. Alex Smith Instructor Associate Professor	Dr. Lisa Robertson Course Coordinator Teaching Staff
Research area	Ecology Biomimetics STEM education	Biodiversity Ecology	Applied invertebrate physiology
Responsibilities	Course content Midterms Exam	Course content Midterms Exam	Online content Assignments Logistics Accommodations Managing teaching and volunteer assistants Grades
Contact hours/week/section	4	4	Highly variable
Means of student contact	Student hours by appointment or open and virtual depending upon course section designation	Student hours by appointment or open and virtual depending upon course section designation	Email to course‐designated address Appointments
Characteristics that were not shared with students			
Times taught the course previously	8	0	6
Previous contributions to course development	Yes	Yes	Yes
Undergraduate teaching experience	8 years	17 years	10 years

### Data collection

At the beginning of the semester, two separate syllabi were distributed, one to each course section, indicating instructor availability for ‘student hours’. Office hours were renamed student hours with the intention of promoting more engagement with students. The syllabi indicated that one instructor would be available 1‐h per week for open, face‐to‐face student hours, while the other instructor would be available by appointment only. The mode of contact for each instructor was reversed between the two sections. The syllabi also informed students that both instructors would be available, separately at different hours for 1‐h per week for virtual student hours, where students could contact them via a ‘chat’ option on the course’s online Learning Management System (LMS). Finally, students were advised that they could contact the teaching team by emailing a course‐designated email address that was monitored by the course coordinator.

Throughout the semester, as students interacted with the teaching team, we collected data on the date of the interaction, the mode of contact (open, appointment, virtual, email), the primary topic of the interaction (selected from a list of predetermined options), the student’s course section, and if it was the student’s first interaction with the instructor. Topic of the interaction included mentorship (e.g., inquiries about career paths or advice for future course selection), accommodation (e.g., inquiries about accommodations for registered disabilities or unexpected health events), favors (e.g., inquiries about relief from an assessment for personal, but not officially recognized, reasons), content learning (e.g., inquiries to better understand a concept) and logistical (inquiries for information about scheduling). For the open, appointment and virtual interactions, the instructors (SJ and MAS) documented each interaction as it occurred. We also collected data from all emails directed to the course‐designated email address. Any emails directed to either instructor pertaining to the course were also forwarded to the course‐designated email address and included in our data collection. Email chains, consisting of an email and one or more responses back and forth on a single issue, were considered a single interaction. Emails where the only purpose was to book an appointment with the instructor were excluded from the data collection. From each email interaction, we then documented which of the teaching team (SJ, MAS, LR) were addressed in the email in addition to extracting data on the previously mentioned variables.

After each class, students typically have 5–10 min to engage with the instructors as they are packing up. In order to capture these interactions, two authors (BEB and VR) attended the end of each class and counted the number of students who approached each of the instructors in the first 5 min after the conclusion of classes each week. The instructors (SJ and MAS) made sure to stand at opposite ends of a large front lectern to ensure that students had to make a choice between the two of them. However, given the number of students and brevity of the interactions occurring after classes, it was not possible to collect any data on the topic of the interaction or the students’ previous interactions with the instructors.

The W20 semester was affected by the global COVID‐19 pandemic. Though many of the ongoing courses ended on March 13, 2020, Discovering Biodiversity shifted to synchronous remote classes and take‐home assessments. Data collection for this study ceased on March 13, 2020, 10 weeks into a 12‐week semester to remove the impact that COVID‐19 would have on our findings.

## Results

**Fig. 1:**
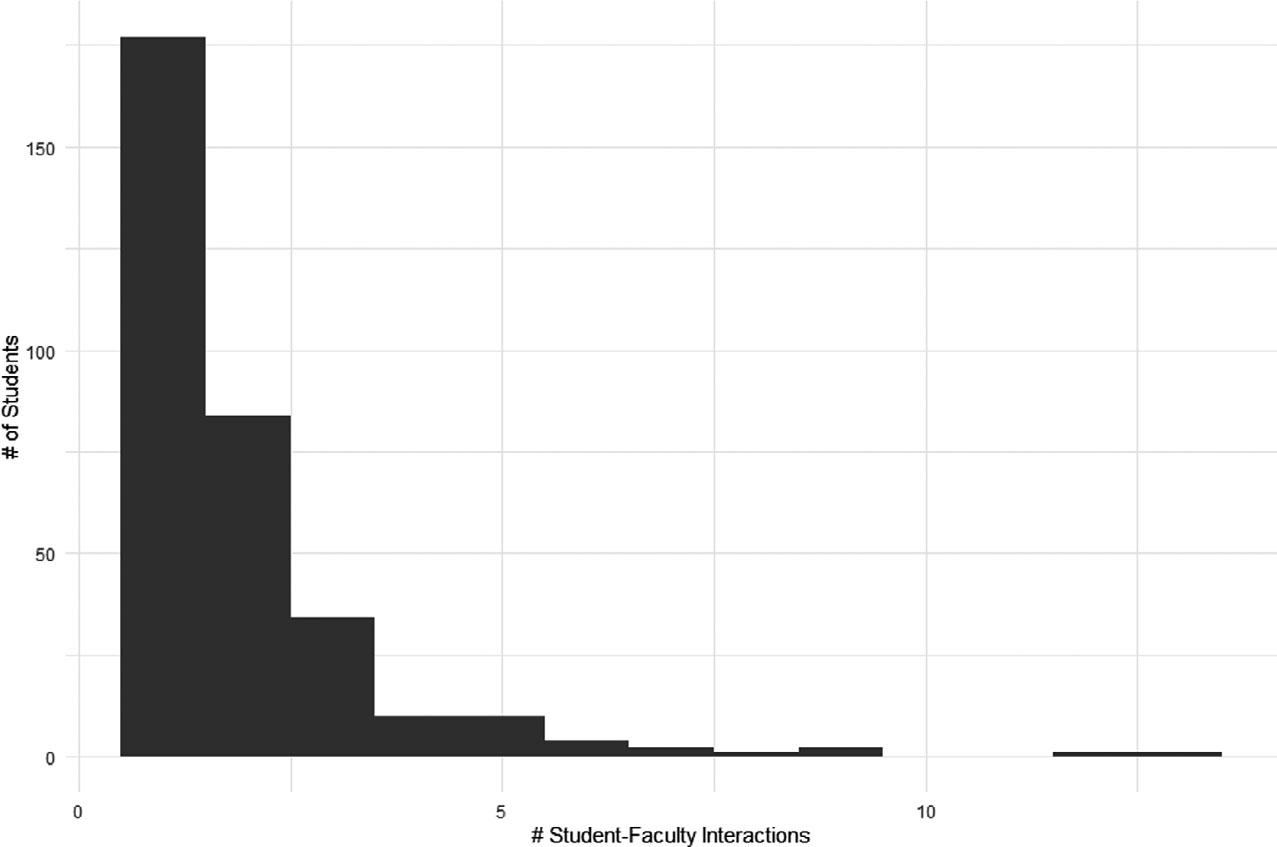
Frequency of the number of interactions that individual students initiated with instructors over the course of the semester. 68.2% of students never made contact with an instructor and are not included in this figure. Of those students who did make contact with an instructor, 54.3% contacted the instructor only once. 3.4% of students contacted an instructor more than five times throughout the semester.

Of the total class size of 885 students between both course sections, 36.8% (*n* = 326 students) made contact with at least one instructor at least once during the semester. This is consistent with previous reports indicating that approximately two‐thirds of students never use office hours [15, 22]. Several of these students contacted the instructor more than once, for a total of 923 instances of contact. Of these, 297 instances of contact were from students approaching instructors after class. Due to the brevity and high number of these interactions, it was not documented whether students who made contact after class had made previous contact with an instructor. Therefore, it is unclear if these instances of contacts were in addition to the 326 students who made contact during other opportunities, or if they were simply previous students returning again. Most students initiated contact with the instructors only once; however, 11 students did contact the instructors more than five times (Fig. 1). Of these 11 students, most interactions were through email and topics mostly consisted of queries about assessment expectations and requests for accommodations as opposed to seeking mentorship or content‐related queries.

### Modes of contact and distribution of conversation topic

**Fig. 2:**
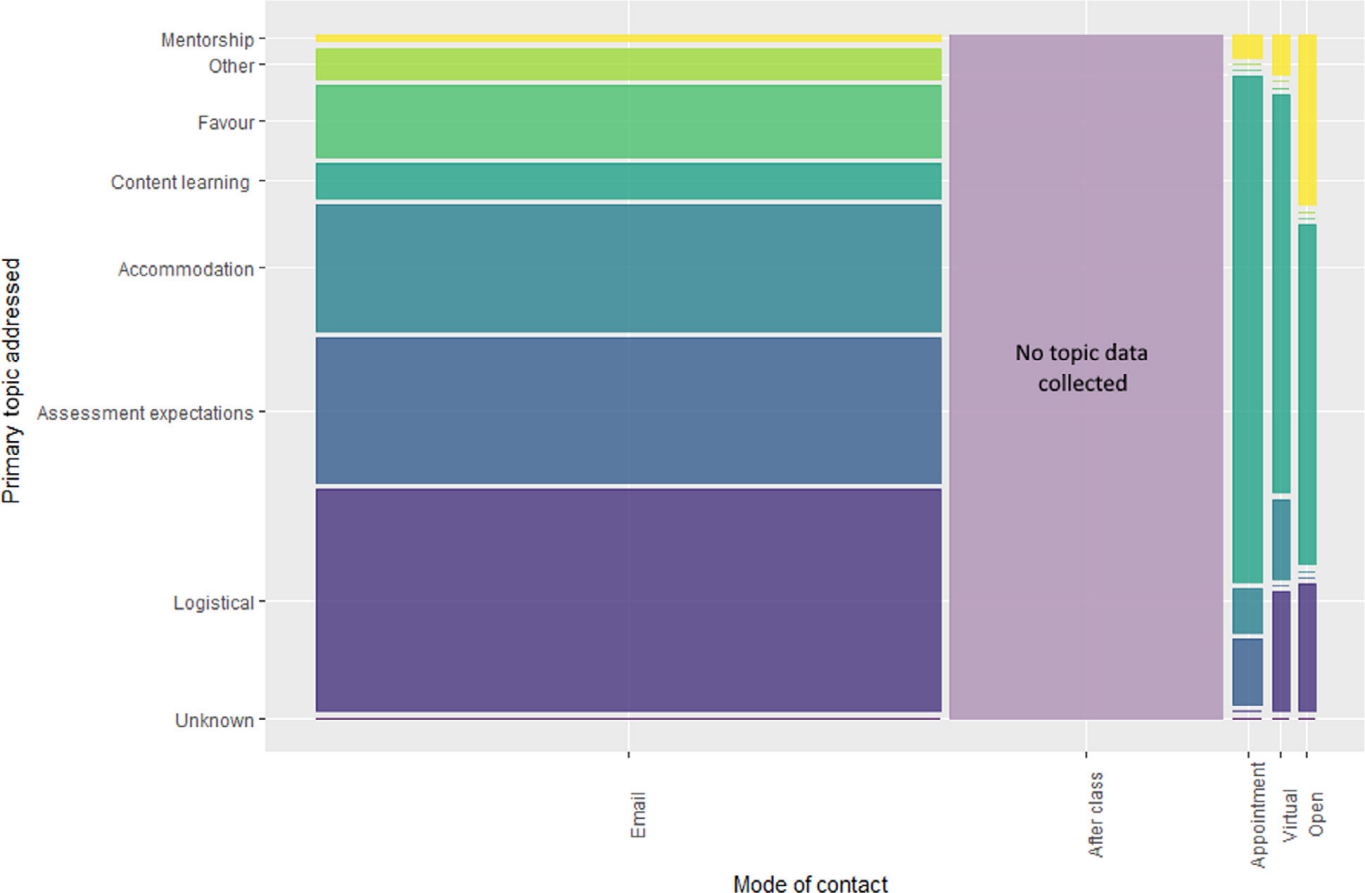
The distribution of topic discussed across each mode of contact. The width of the bars corresponds to the relative number of contacts made within each mode. Given the brevity and quantity of interactions after class, we were unable to collect topic data for this modality.

The greatest number of student–instructor interactions were facilitated through email and through interactions with instructors after class. A total of 601 email conversations were initiated by students, constituting 65.1% of the total interactions with the instructors throughout the semester. Though emails accounted for most of the interactions between instructors and students, the majority of the email topics were inquiries about course logistics, asking for favors and clarifying assessment expectations (Fig. 2). Content‐related interactions accounted for 5% of the total email interactions. Conversations after class were the second most frequent form of student–instructor contact, making up 28.4% of the total instances. Due to the brevity and the lack of time to record between these conversations after class, it was not possible to collect data on the topic of these interactions. However, based on instructor reflection, we recollect that the majority of these questions were brief clarifying questions related to course logistics and content.

The remaining 6.5% of interactions were conducted through one of the three ‘student hour’ styles (by appointment: *n* = 29; open student hours: *n* = 15; virtual student hours: *n* = 16). While the student hour interactions represented the smallest proportion of student–instructor interactions in our study, the topics discussed tended to be the most diverse. Furthermore, the range of topics discussed was different between the three modes. While content learning was the most common topic of discussion across all three modes, this trend was most pronounced in the by‐appointment interactions, where content learning accounted for 79% of these interactions as compared to 53% during open student hours. Comparatively, mentoring occurred most frequently in open student hours (26% as compared to 3% in appointments). While both the appointment and open modes of student hours included some logistical topics, organizational interactions during appointments primarily focused on clarifying assessment expectations and arranging accommodations, whereas organizational topics regarding logistics were the focus in the open student hour sessions. Taken together these results indicate value to both the appointment and open styles of student hours. Students may interpret open student hours as a more casual format for brief questions or genuine conversation, whereas booked appointments may be interpreted as more serious, solution‐oriented sessions. Future research should explore these differences through interviews or other rich qualitative methods.

The topics discussed in the third mode of student hours, virtual student hours, were very similar in distribution to both the open and by‐appointment styles. Predominant topics included content learning, mentorship, arranging accommodations, and logistical questions, suggesting that virtual student hours are sufficient to support all topics of conversation as do in‐person student hours. Additionally, the total number of interactions initiated in virtual student hours was comparable to that of both in‐person formats. Virtual student hours appeared to elicit conversations similar to those of the other student hour styles compared to email interactions. This is likely due to the ‘instant messaging’ format of the virtual student hours, which allows for the same ‘back‐and‐forth’ that is associated with in‐person student hours. This finding may have important implications for virtual learning in the wake of the COVID‐19 pandemic.

### Topic of interaction changes around assessment time

**Fig. 3:**
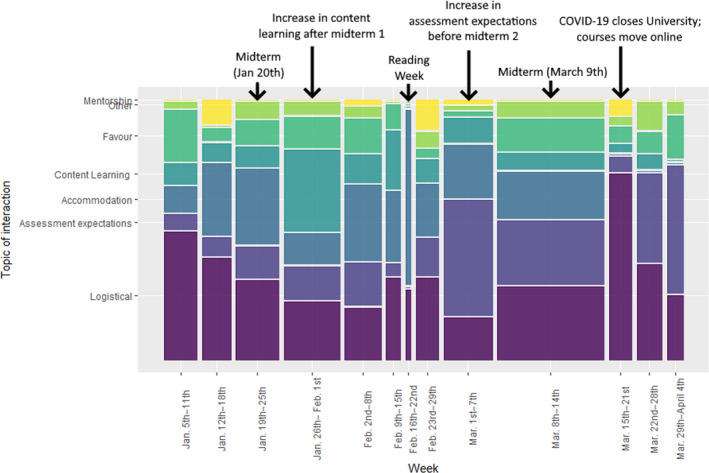
The distribution of topics discussed during student interactions with instructors throughout the semester by week. The width of the bars corresponds the relative number of interactions within each week. Notable events which occurred during the semester are included in black.

Topics discussed throughout the semester follow a predictable pattern. Mentorship, favors, and topics categorized as ‘other’ are relatively evenly distributed throughout the semester. However, there are two major changes in distribution of topics to note, both regarding midterm examinations. Firstly, there was a substantial increase in contact immediately following the first midterm (Fig. 3). Much of this increase can be attributed to a rise in queries related to content learning. Secondly, there was an increase in the number of students asking about assessment expectations before the second midterm. There was also an increased proportion of logistical queries in the third last week of the semester, which was likely due to the rapid shift to virtual learning and instruction from face‐to‐face instruction on March 13th, 2020 due to the COVID‐19 pandemic (Fig. 3).

Given the increased contact surrounding each of the major assessments in this course, it may be particularly important to ensure that more student hours are available during these periods. Furthermore, given the increased proportion of queries related to content learning and assessment expectations, it may be beneficial to advertise the instructors’ availability by both appointment and during scheduled student hours to ensure that all students are provided ample opportunity to interact with instructors.

### Personal characteristics of instructor impact frequency and topic of student–instructor interactions

**Fig. 4:**
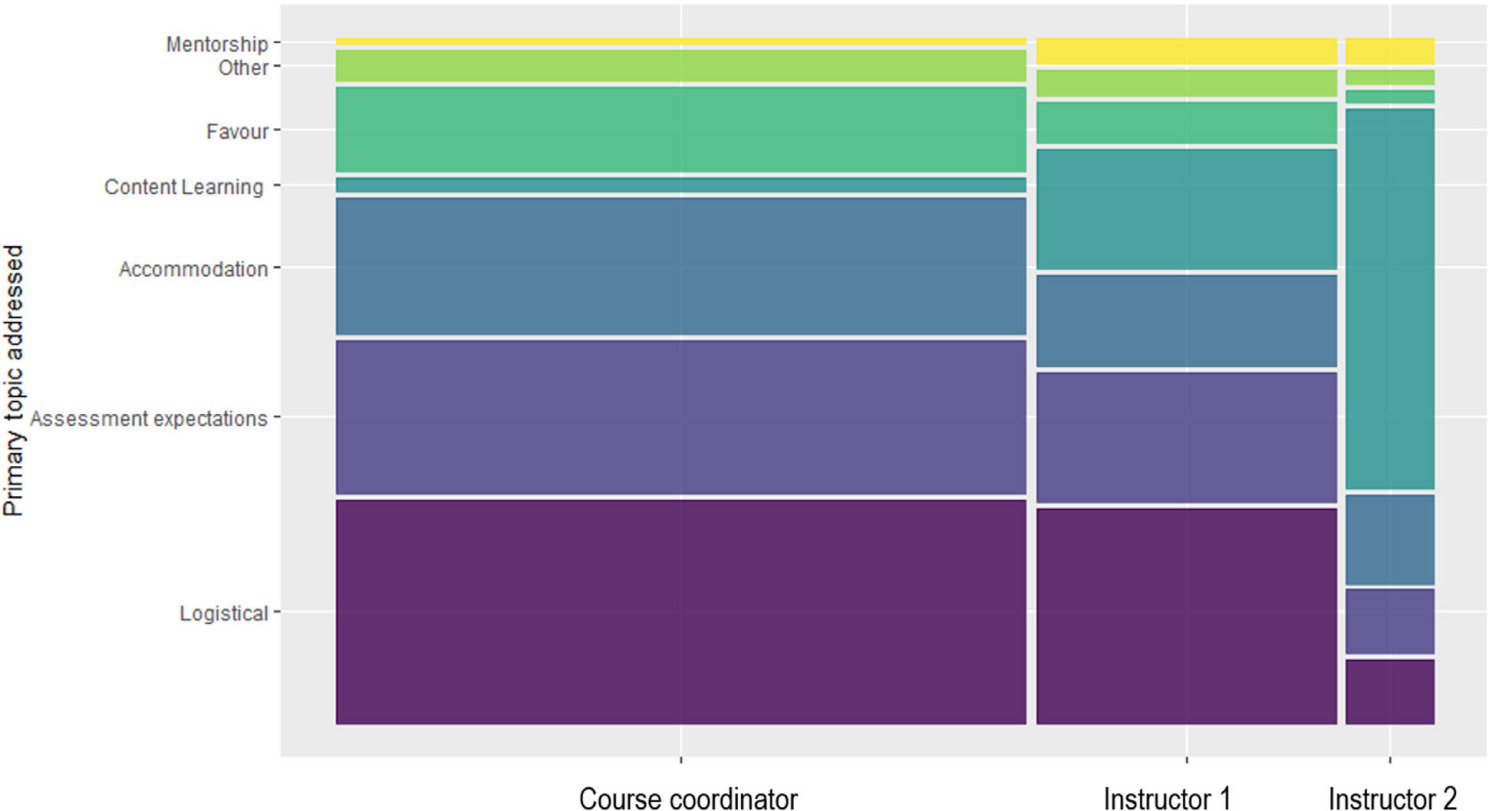
The distribution of topics discussed during interactions with each member of the teaching team, including the course coordinator. The width of the bars corresponds to the proportion of the total number of interactions initiated by students toward each member of the teaching team. The course coordinator interactions with students occurred entirely by email, while the interactions between students and the instructors occurred through email, after class, virtual office hours, in‐person office hours, and appointments.

Though more students approached instructor 1 after class and for student hour style interactions at a higher frequency than instructor 2, these differences were not significantly different from a 50 : 50 ratio of interaction (instructor 1 : 56.9%, *n* = 149; instructor 2 : 43.1%, *n* = 113; X_2_ = 2.46, *P* = 0.11). However, over the course of the semester, 25.0% of emails were addressed solely to the instructor 1 (*n* = 150), while only 3.2% of emails were addressed solely to instructor 2 (*n* = 19), which was a significant increase from a 50 : 50 ratio (X_2_ = 59.9, *P* =< 0.001) and were most often sent to their personal addresses despite instructions to use the course‐designated email address. An additional 2.8% of emails were addressed to both of the instructors (*n* = 17). The final 69.1% of the emails (*n* = 415) were either unaddressed, addressed to the course coordinator, or addressed to a combination of teaching staff that included at least one instructor and the course coordinator. The topics discussed with each of the instructors also differed substantially (Fig. 4). Instructor 1 received far more logistical queries and the distribution of topics discussed more closely resembled that of the course coordinator than that of instructor 2. Indeed, 59% of instructor 2’s interactions with students had to do directly with course content, whereas this was true of only 18% of the interactions between students and instructor 1. These results indicate that students are not only more likely to favor the same instructor of a teaching team with logistical questions, but that there is a higher workload with respect to number of interactions (predominantly by email). These results align with a wealth of research outlining the disparities in workload of instructors depending upon their perceptible characteristics [35.36].

## Discussion

The aim of this study was to investigate how students choose to engage with different formats of student–instructor contact, in order to devise best practices for supporting the greatest level of high‐quality student–instructor contact. Overall, appointments seem to result in more content‐oriented student–instructor interaction; however, open student hours allowed students to drop by with pressing logistical questions and was most commonly where mentoring occurred. It is important to note that, apart from email, the greatest number of students interacted with instructors directly after class, which supports previous research indicating student preference for this format of contact [20]. We are not able to draw conclusions about the depth of these interactions, as we did not collect topic data; however, past research has indicated that these brief interactions pose little benefit to students [19]. Instructor reflection indicates that these interactions were mostly related to logistical questions, assessment expectations, and clarifying of course content. Regardless, this may be the only face‐to‐face contact that many students have with their instructors and it therefore warrants additional research into how to facilitate meaningful conversation during these interactions.

Our study supports previous observations of low incidence of student–instructor contact outside of class time that has been documented in past research [18, 19, 20, 21]. Specifically, our data indicate that almost two‐thirds of the students never contacted instructors in any form of student hours or by email, aligning with student self‐reported data indicating that two‐thirds of students never used office hours [15, 22]. This finding is particularly notable, given that our study offered diverse options for making contact with the instructors, which aimed to alleviate commonly cited barriers such as lack of convenience and instructor availability [15, 22, 23, 24]. The continued disengagement with office hours shown in our study, despite convenient and adaptable formats, may be attributed to past findings of a mismatch between students’ perception and the intended use of office hours [15]. We speculate that after‐class interactions may be perceived as more informal and lower‐stakes by students, supporting increased engagement. Therefore, we recommend the development of policies and practices that allow for higher‐quality contact to occur in the time directly after class. For example, the classroom could be booked for additional time after class to empower students to spend more time discussing substantive topics with the instructor. Or areas immediately outside of classrooms could be co‐opted for such interactions, to ensure that interactions are less rushed and do not interfere with the next use of the classroom space.

Understanding the pattern of student interactions over the course of the semester is another valuable tool for ensuring that instructors are available to students when they are needed the most. Our study revealed a pattern of student interactions over the ten weeks of our twelve‐week course, where the greatest number of interactions occurred immediately after the first major assessment and prior to the second. Specifically, after the first course midterm, students initiated more contact and primarily discussed content learning. Presumably, this is representative of students aiming to strengthen their knowledge in areas where they did not do well in the first assessment. Prior to the second major assessment, there was an increase in discussion related to assessment expectations. These interactions may represent the students aiming to gain clarity on how to improve before the second assessment. We found that student hours booked by appointment are more likely to result in discussion of assessment expectations and therefore should be made available to students at least, if not especially, prior to major assessments. This could be available in addition to open and virtual student hours, which are comparable in their promotion of content learning. These recommendations could also be combined with our previous recommendation by providing increased availability after class in the time directly before and after major assessments.

Another finding of this study was the pronounced difference in patterns of contact between the two instructors. This has implications for supporting the diversification of teaching teams when we aim to increase student engagement. Our study showed students initiated face‐to‐face contact with one instructor at higher rates than the other instructor. Additionally, the topics discussed were markedly different between instructors, indicating that students may initiate contact for different topics conditionally, based on shared preferences. This aligns with previous findings showing patterns in how students interact with instructors with different characteristics [32, 33]. Specifically, students opted to contact instructor 1 with a far greater number of queries related to course logistics and accommodations, despite there being a designated course coordinator responsible for these matters. We cannot be certain why students were choosing to contact different instructors for different reasons though we do suggest here that sometimes, the formally presented reason for contact may not be the actual reason. For example, many of the interactions with students may have begun with the answering of a logistical question but ended with conversation about mental health or well‐being.

While the implications of diversity in teaching teams on student learning should be considered, this finding has important implications for teaching administration. In our study, the breakdown of topics discussed with instructor 1 was markedly similar to those discussed with the course coordinator and quite dissimilar to what students discussed with instructor 2. Additionally, instructor 1 received approximately eight times as many emails from students as instructor 2 over the course of the semester. This finding aligns with past research showing that students find female‐presenting instructors more approachable [31] and are therefore more likely to contact female instructors [32], creating a greater, and often invisible, workload for these instructors [33]. Given that both instructors in this study were rated similarly on their student evaluations, it is unclear how approachability impacts our findings, though it is evident that instructor 1 was approached more often. However, it is important to note that instructor 1 has taught the course more times than instructor 2 and while this information was not shared with students, it is possible that this gave instructor 1 a reputation among first‐year students that instructor 2 was not afforded. Nonetheless, our findings indicate that the greater burden of work for the instructor contacted more frequently is hidden from their colleagues in the form of emails, creating the potential for this burden to be overlooked and unaccounted for either because of their characteristics or because they are the preferred instructor in a teaching team. This additional work should be recognized and accounted for by the administration.

## Recommendations

Recommendations for policy to promote meaningful interactions between instructors and students and help achieve work‐equity are below.

### Recommendations to promote more accessible student–instructor interaction

Students prefer to use after class time to speak with instructors. While these times may not allow for meaningful interactions, they are opportunities to engage with students and demonstrate that interactions are welcome. Leaving time available to facilitate student questions after class will not only promote more student interactions, it would reduce the number of unanswered questions that would likely be directed to email. The following suggestions aim to make contact time more accessible:

1. Make time immediately after class where students do not have to modality‐shift
  - Stand outside of lecture hall after class;
  - End class early to extend ‘after class’ conversation;
  - Make a remote student hour immediately following a synchronous remote class.
2. Schedule regular student emailing windows within the day so students can expect timely responses to questions.
3. Further clarify assessment expectations for all students when responding to a specific query to reduce repeated questions and allow all students to benefit from clarification.

### Administrative recommendations

The burden should not be on instructors, especially those most likely to be contacted by students, to offer a lesser educational service to students due to disparities in actual contact time among instructors in teaching teams. Doing so places the responsibility upon instructors to choose between an increase in workload or offering a lesser teaching experience that could harm their career progression. Administrators need to build within their systems the recognition that instructor differences in workload exist and that student–instructor contact outside of class are valuable educational opportunities. As such, support should be provided to instructors from equity‐seeking communities in a tangible way. Examples include:

1. Redistribute workload across teaching tearms and instructors in the department in recognition of the increased workload for equity‐seeking instructors.
2. If in a teaching team: recognize that most of the individual contact time with students will be done by one of the instructors and assign more logistical or development work to the other instructor. (Use the extensive published research on the topic to determine which instructor is likely to be contacted most.) These other activities might include the writing of exams or assignments, preparation of teaching materials, or grading.
3. If instructors are not team teaching: recognize that some instructors (e.g., from equity‐seeking communities) are more likely to have more individual contact time with students by increasing the weight that each course has, especially large courses, on their workload distribution.

## Conflict of interest

The authors declare no conflict of interest.

## Author contributions

All authors participated in the data collection as described in the methods and all authors participated in the design. BEB and VR analyzed the data and wrote the first draft and SJ, LR, and MAS provided revisions. SJ conceived of the study.

## Data Availability

Due to the terms of the University of Guelph Research Ethics Board (REB# 19‐05‐022), for a deceptive study where consent was not sought, the data are available only by request to the corresponding author. Video data are not available.
